# Emergency Medical Services Calls for Service at Adult Detention Centers: A Descriptive Study

**DOI:** 10.5811/westjem.33613

**Published:** 2025-07-12

**Authors:** Jeffrey N. Wood, Aaron B. Klassen, Matthew D. Sztajnkrycer

**Affiliations:** Mayo Clinic, Division of Prehospital Care, Department of Emergency Medicine, Rochester, Minnesota

## Abstract

**Introduction:**

Incarcerated individuals represent a vulnerable sector of society, with a disproportionate burden of substance use, mental health problems, and chronic illness. The purpe of this study was to perform a descriptive analysis of emergency medical services (EMS) response to detention facilities.

**Methods:**

We conducted a retrospective review of Mayo Clinic Ambulance Service ground EMS emergency (9-1-1) calls for service to nine detention centers within the service area occurring between January 1, 2002–December 31,2021. We excluded calls to a 10^th^ detention center, the Federal Medical Center – Rochester, due to the unique nature of this facility. Additional exclusion criteria included non-emergency calls and lack of patient care narratives within the patient care report. We analyzed data using descriptive statistics, chi-square, and the Student *t*-test. This study was reviewed and approved by the Mayo Clinic Institutional Review Board.

**Results:**

During the study period, 3,114/1,231,853 (0.25%) service requests to detention facilities occurred. After accounting for exclusion criteria, the final sample size consisted of 2,034 patients. Average patient age was 40.2 ± 13.3 years of age, compared with 54.0 ± 25.9 years of age for non-detention center calls (*P* < 0.001). The majority (80.8%) of patients were male. Mean scene time was 14:13 ± 7:49 minutes, compared with 12:04 ± 12:27 minutes (*P* < 0.01) for non-detention center calls. The most common complaints were medical, behavioral emergencies, cardiac, and trauma. Obstetrics requests accounted for 5.8% of calls for female patients. Most calls (91.3%) to detention centers involved incarcerated individuals, with the remainder representing facility staff (1.5%), visitors (0.5%), and undetermined (6.7%). Nearly 4% of patients refused treatment; 48.9% of these patients were still transported. Consent for treatment/transport by the patient was documented in 6.1% of charts.

**Conclusion:**

Recognizing the retrospective, single-agency nature of this study, we found that calls to detention facilities within our 9-1-1 service area predominantly involved incarcerated individuals. Consent for treatment/transport was not documented in most EMS encounters. Further study is needed to better understand the healthcare needs of these patients, including ability to consent.

## INTRODUCTION

The United States (US) has the highest reported prison population in the world, and the highest incarceration rate in the western world.^1^ In 2008, 1 in every 100 US adults was behind bars.^2^ Incarceration rates among minority populations were even more stark; 1 in 36 Hispanic males ≥18 years of age was incarcerated, as was 1 in 15 Black males. Incarcerated individuals represent a vulnerable sector of society, and incarceration itself forms a social determinant of health.^3^,^4^ Studies suggest that up to 76% of incarcerated adult males have substance use and/or mental health disorders.^5^–^7^ These individuals also have a disproportionate burden of chronic illness compared with the general public, including heart disease, cancer, and HIV.^8^

Deaths in detention facilities are increasing.^9^ Suicide is the single leading cause of death, accounting for approximately 30% of all prisoner deaths.^10^ However, 46% of deaths are due to illness, including heart disease, liver disease, and cancer. The number of deaths due to substance intoxication quadrupled between 2000 and 2018.^9^ COVID-19 incidence and standardized mortality were higher in prisons than in the general US population.^11^

Incarcerated and recently released individuals are frequent users of emergency departments.^12^–^15^ Despite this, little is known about the emergency medical services (EMS) response to detention facilities.^16^–^18^ A recent news report highlighted the issue of delayed EMS access to incarcerated patients resulting in death.^19^ One EMS article suggested a deceptive agenda for EMS use by incarcerated individuals, using the pejorative term ”incarceritis” to suggest malingering and inappropriate transport.^17^

### Purpose

Given this identified knowledge gap surrounding a vulnerable patient population, our goal in this study was to perform a descriptive analysis of EMS response to detention centers to better understand the nature of patients (eg, incarcerated individual, facility staff, visitor) and associated complaints and, therefore, the operational needs and training requirements for our EMS agency, as well as to identify the unique patient care needs of this population.

## METHODS

We conducted a retrospective review of all EMS calls for service to detention facilities served by a single EMS agency between January 1, 2002–December 31, 2021. The study was reviewed and approved by the Mayo Clinic Institutional Review Board.

### Study Setting

Mayo Clinic Ambulance Service (MCAS) is a comprehensive prehospital care system, including ground EMS and helicopter EMS assets. MCAS is the sole Advanced Life Support ground transport service for the served areas, with 18 ambulance bases covering 6,894 square miles responsible for providing both 9-1-1 response and interfacility transportation. The service also provides emergency intercept for regional Basic Life Support services. Within the service area are 10 detention facilities: seven county jails; one state prison; and two federal prisons, one of which is the Federal Medical Center (FMC) - Rochester. Jails and prisons differ in terms of populations and resources. Jails are short-term municipal facilities, used for those newly in custody, those awaiting trial or sentencing, and those sentenced to serve custodial sentences <1 year. In contrast, prisons are state or federal institutions in which convicted offenders serve sentences >1 year.

**Population Health Research Capsule**

What do we already know about this issue?

*Although incarcerated individuals represent a vulnerable population, very little is known about their medical needs requiring EMS 911 assessment*.

What was the research question?

*To perform a descriptive analysis of a single EMS agency’s response to detention centers*

What was the major finding of the study?

*Most patients (91.3%) were incarcerated. Consent for treatment/transport was documented in only 6.1% of charts*.

How does this improve population health?

*Behavioral health emergencies are most common in jails, providing an opportunity for collaborative interventions. Further study is needed to better understand barriers to consent*.

### Study Design

We electronically abstracted all MCAS emergency (9-1-1) calls for service to an adult detention facility based upon service address/name from stored electronic patient care reports (ePCR) into a de novo, deidentified Microsoft Excel for Mac 2023, v16.77.1 (Microsoft Corporation, Redmond, WA) data collection instrument. Data points included facility name, time call originated, at-scene time, transport time, age, sex, transport priority, chief complaint, vital signs, interventions performed, transport outcome, and patient narrative record. Although the study was primarily a descriptive analysis, we abstracted the data and reported it in accordance with the Strengthening the Reporting of Observational Studies in Epidemiology (STROBE) checklist, using best practices for retrospective chart reviews.^20^–^22^

Exclusion criteria included non-emergency calls and calls for service at the FMC - Rochester, as these calls represent interfacility transfers rather than primary emergency responses. Although data points other than the narrative record were analyzed, we excluded from review calls with no narrative recorded from the final dataset as lack of narrative precluded assessment of consent and transport outcomes. Individual EMS patient care reports were reviewed to ensure that the final cohort of cases met inclusion and exclusion criteria.

### Data Analysis

Using Microsoft Excel, we summarized numeric data with means and standard deviations; categorical data were summarized with frequency counts and percentages. Data were analyzed using descriptive statistics. We compared patient characteristics using a two-sided Fisher exact test and unpaired two-sample *t*-tests. *P*-values less than 0.05 were considered significant.

## RESULTS

During the study period, EMS responded to 1,231,853 emergency (9-1-1) response calls for service, of which 3,114 (0.25%) were calls to detention facilities. Of these, 138 did not involve EMS patient contact, and 942 had no associated patient narrative, resulting in a final cohort of 2,034 patients. Patient demographics are provided in Table 1. Mean scene time for detention center calls was 14:13 ± 7:49 minutes, compared with 12:04 ± 12:27 minutes (*P* < .01) for all other non-detention center 9-1-1 calls. Forty-nine calls explicitly documented extended delays accessing the patient due to the nature of the facility. Facility medical personnel were present prior to EMS arrival in 383 (18.8%) cases. State and federal prisons were more likely to have facility medical personnel (43.2%) than county jails (11.8%, *P* < .01). The most common chief complaints are listed in Table 1 and Table 2. Obstetrics (OB) requests accounted for 5.8% of calls for female patients. Behavioral emergencies and overdoses were more common in individuals incarcerated in county jails (370 and 50, respectively, vs 36 and 5) while cardiac issues were more common in state and federal prisons (122 vs 171). Differences in chief complaints were noted between inmates, facility staff, and visitors (Table 2). In 2% of cases, EMS personnel were called and asked to provide medical clearance. Within the ePCR narratives, documentation of consent for treatment and patients’ wishes was infrequent (Table 3). Despite treatment refusal by 4.6% of patients, 42.5% of these patients were transported, all of whom were incarcerated. Treatment was specifically requested by 2.7% of patients; this was denied in 0.5% of patients. Compared with staff and visitors, inmates were more likely to be refused transport (*P* = .21) or transported against their explicit wishes (*P* < .001; Table 3). Sample narratives surrounding consent are provided in Table 4.

## DISCUSSION

In the current study, most patient encounters involved inmates (91.3%, Table 1, Table 2). Incarcerated populations frequently over-represent minorities, have higher rates of substance use disorder and mental illness than the general population, and a limited ability to access the emergency medical care system.^2^–^7^ Incarceration itself may, therefore, be viewed as a social determinant of health.^4^ Literature regarding EMS management of incarcerated patients is sparse and often explicitly biased against this group.^17^,^24^ Based upon ePCR narratives, EMS personnel in the current study occasionally demonstrated both explicit bias and confusion regarding an inmate’s ability to both access and refuse treatment (Table 4). Most US case law in this area involves violations of the Eighth Amendment of the US Constitution, which forbids cruel or unusual punishment. Two specific cases, Estelle v Gamble and Farmer v Brennan, are frequently cited but are focused on deprivation rather than the refusal of care. 25,26 Neither ruling addresses medical decision-making by prisoners. Two decisions, Quinlan and Cruzan v Director, Missouri Department of Health, provide everyone, including competent prisoners, with the right to self-determination, including the right to refuse treatment.^27^,^28^,^29^ Despite this, consent was rarely documented in the patient care report. Although occurring infrequently, inmates were both refused transport and transported against their explicit wishes.

In contrast to the general population, restrictions placed upon access to inmates may serve to delay EMS response. The ePCR narratives specifically identified delays in patient access in 49 cases; therefore, EMS agencies should be aware of the logistical constraints in responding to calls for service at custodial facilities. Most patients in this study were detained in local jails. This may result in differences both in patient populations and medical complaints (Table 1). In contrast to prisons, which often have medical facilities on site, jails are less likely to have these resources. EMS agencies serving communities with detention facilities should plan accordingly. Differences in patient complaints were also noted based upon the nature of the patient (inmate, staff, visitor) (Table 2).

Due to their short-term nature, jail populations tend to be younger than those in prisons. In the current study, jail populations had an age of 38.43 ± 12.03 years, state prison populations had an age of 39.12 ± 12.75 years, and federal prison populations 52.96 ± 13.44 years. As previously noted, overdose and behavioral complaints were more common in jails.^6^.^30^ This may reflect the fact that jail populations, being younger and often incarcerated for short periods of time, have difficulty adjusting to custodial sentences. Jail tends to be more unpredictable than prison, resulting in increased perceived stress.^31^ Alternatively, jail detainees may still be suffering from the acute effects of substance exposure; thus, this population may be more likely to include patients with underlying behavioral health conditions resulting in their incarceration, which in turn may be less likely to result in felony conviction and prison sentences. Regardless, the prevalence of behavioral health calls to jails may provide an opportunity both for facility-based and EMS-based behavioral health crisis intervention teams.

Females represented 20.1% of the population in this study. The majority were encountered in jail settings compared with state or federal prisons. Only four calls for service in federal prisons involved female patients. One hundred and thirty-six calls for service at jails involved OB-related complaints.

Emercency medical services were requested to respond to custodial facilities to perform medical screening evaluations in 2% of cases. Although an uncommon occurrence, these are high-risk patient encounters. EMS agencies should consider policies and protocols for these requests, as well as perform quality assurance on all these calls.

## LIMITATIONS

This study was subject to several limitations. As with any retrospective analysis, it was prone to biases, including selection bias and misclassification bias. Initial patient stratification was based upon dispatch to detention facilities. Many jails, however, are part of larger municipal complexes. We excluded 942 from the final study cohort due to lack of patient care report narratives, representing 30.3% of the initial dataset and potentially biasing the analysis. Only a single EMS system was evaluated. Each EMS system is unique and should be viewed as such. The geography and patient complaints noted in our study may not be generalizable to other systems.

A large federal prison located within the study area was excluded from this analysis. FMC - Rochester is one of seven federal Bureau of Prisons medical referral centers that provide specialized medical care and function as a medical prison.^32^ However, the advanced medical care available at FMC - Rochester means that it is fundamentally different from other custodial facilities, serving as a healthcare facility for the federal prison population. Patients are only transferred when in need of advanced diagnostic workups, specialist assessment, or higher levels of care.

Consent for treatment and transport was rarely noted in the patient narrative. It may be that crews obtained consent but simply did not document this. The rate of consent documentation in other patient populations within this EMS system is unknown. Consent may also be implied by the fact that an inmate specifically requested medical evaluation. Based upon the medical narrative, it was not always clear who initiated the request for EMS response. Due to the retrospective nature of the study, it is also unclear why consent was explicitly documented in 7.0% of cases.

## CONCLUSION

Within our 9-1-1 service area, calls to detention facilities occur at a low frequency. Behavioral health emergencies are most common in county jails, providing an opportunity for collaborative interventions. Consent for treatment/transport was not documented in most EMS encounters. Although infrequent, inmates are both more likely to be transported despite refusal and to be refused transport despite requesting emergency department evaluation when compared with staff and visitors. Further study is needed to better understand the health care needs of these patients, including ability to consent.

## Figures and Tables

**Table 1:** Patient characteristics based upon level of incarceration in a study of emergency medical services calls to adult detention centers.

	All	Jail	State prison	Federal prison
Total Patients (n)	2,034	1,480	341	213
Age (Y)	40.1 ± 13.1	38.4 ± 12.0	39.1 ± 12.8	53.0 ± 13.4
Male (%)	79.9	73.5	95.6	98.2
Chief complaint (%)				
Behavioral	20.6	26.0	9.8	1.4
Cardiac	14.9	12.0	20.1	26.2
Medical	43.5	41.9	45.1	52.3
Obstetrics	1.5	2.0	0.0	0.5
Poisoning/overdose	3.0	3.5	1.5	0.0
Respiratory	5.2	3.4	10.7	8.6
Trauma	11.3	11.2	12.8	11.0
Patient type (%)				
Inmate	91.3	93.1	88.0	80.3
Staff	1.5	1.2	3.5	0.5
Visitor	0.5	0.0	1.5	0.9
Undetermined	6.7	5.7	7.0	18.3
Transport Mode (%)				
Ground	100	100	100	100
Air	0	0	0	0
Transport Priority (%)				
Priority 1 (L+S)	2.6	1.6	11.8	1.9
Priority 2 (No L+S)	97.4	98.4	88.2	98.1

*L+S*, lights and sirens.

**Table 2:** Selected patient complaints based upon patient status in a study of emergency medical services calls to adult detention centers.

	All	Inmate	Staff	Visitor
Total Patients (n)	1,833	1,779	26	28
Behavioral	21.7%	21.8%	0.0%	17.9%
Cardiac	14.2%	14.2%	23.1%	14.3%
Medical	43.8%	43.6%	53.8%	57.1%
Obstetrics	1.3%	1.3%	0.0%	0.0%
Poisoning/overdose	2.9%	3.0%	0.0%	0.0%
Respiratory	4.9%	5.0%	3.8%	0.0%
Trauma	11.2%	11.1%	19.3%	10.7%

**Table 3:** Patient incarceration status and transport outcomes in a study of emergency medical services calls to adult detention centers.

	Inmate	Staff	Visitor
Patient Mental Status as Documented in the ePCR			
Altered mental status	309 (18.6%)	0 (0%)	3 (20%)
Not noted	1,257 (75.8%)	22 (100%)	12 (80.0%)
Uncooperative	92 (5.6%)	0 (0%)	0 (0%)
Patient Transport Request and Subsequent Disposition			
Request + transport	35 (29.4%)	1 (16.7%)	6 (46.2%)
Request + no transport	9 (7.6%)	0 (0%)	0 (0%)
Refuse + transport	37 (31.1%)	0 (0%)	0 (0%)
Refuse + no transport	38 (31.9%)	5 (83.3%)	7 (53.8%)

*ePCR*, electronic patient care report.

**Table 4:** Selected patient care report narratives in a study of emergency medical services calls to adult detention centers.

The pt argued for a while that he did not want to go to the hospital but eventually gave into her wishes.
Patient was in custody and treated and transported under the authority of the [Redacted] Correctional Facility
COULD NOT OBTAIN SIGNATURE BECAUSE PATIENT WAS PRETENDING TO HAVE AN ALTERED LEVEL OF CONSCIOUSNESS.
Patient was refusing care, but [Redacted] County policy states that patient must be checked out at emergency department. Patient refused signature
Did not want to go to the hospital. Patient finally decided to cooperate, as deputies advised that he was going to have to go regardless of the situation, due to him being in custody
Did not wish to be transported, however she was not able to decide because she was in custody
We came to a conclusion that the pt. had no head injury or need for further medical treatment.
PT stated that she did not want to go to the hospital. Jail personnel advised the PT that she did not have a choice in the matter and that she would be transported for evaluation.

*pt*, patient.
